# 7-Methyl-9-*p*-tolyl-4,9-dihydro­furo[3,4-*b*]quinolin-1(3*H*)-one

**DOI:** 10.1107/S1600536808041457

**Published:** 2008-12-13

**Authors:** Chunling Shi, Min Ji

**Affiliations:** a School of Chemistry and Chemical Engineering, Institute of Pharmaceutical Engineering, Southeast University, Nanjing 210096, People’s Republic of China

## Abstract

In the title compound, C_19_H_17_NO_2_, the dihydro­pyridine ring adopts a flattened boat conformation while the furan­one ring is almost planar (r.m.s. deviation 0.018 Å). The mol­ecules are linked into chains along the *b* axis by N—H⋯O inter­molecular hydrogen bonds. In addition, C—H⋯π inter­actions involving the phenyl ring of the tolyl group as π acceptor are observed.

## Related literature

For the biological activities of podophyllotoxin and its derivatives, see: Bosmans *et al.* (1989 ▶); Eycken *et al.* (1989 ▶); Hitosuyanagi *et al.* (1997 ▶, 1999 ▶); Lienard *et al.* (1991 ▶); Magedov *et al.* (2007 ▶); Poli & Giambastiani (2002 ▶); Tomioka *et al.* (1989 ▶, 1993 ▶); Tratrat *et al.* (2002 ▶).
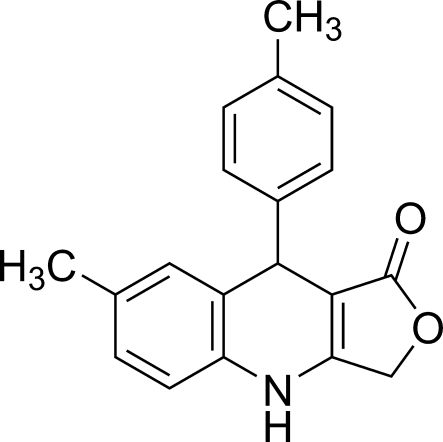


## Experimental

### 

#### Crystal data

C_19_H_17_NO_2_
*M* *_r_* = 291.34
Monoclinic, 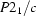

*a* = 9.178 (2) Å
*b* = 11.457 (2) Å
*c* = 14.350 (4) Å
β = 103.124 (5)°
*V* = 1469.5 (6) Å^3^
*Z* = 4
Mo *K*α radiation
μ = 0.09 mm^−1^
*T* = 223 (2) K
0.60 × 0.48 × 0.45 mm

#### Data collection

Rigaku Mercury diffractometer
Absorption correction: multi-scan (Jacobson, 1998 ▶) *T*_min_ = 0.756, *T*_max_ = 0.962
13947 measured reflections
2675 independent reflections
2364 reflections with *I* > 2σ(*I*)
*R*_int_ = 0.034

#### Refinement

*R*[*F* ^2^ > 2σ(*F* ^2^)] = 0.059
*wR*(*F* ^2^) = 0.135
*S* = 1.16
2675 reflections
202 parameters
H-atom parameters constrained
Δρ_max_ = 0.23 e Å^−3^
Δρ_min_ = −0.21 e Å^−3^

### 

Data collection: *CrystalClear* (Rigaku, 2000 ▶); cell refinement: *CrystalClear*; data reduction: *CrystalStructure* (Rigaku/MSC, 2003 ▶); program(s) used to solve structure: *SHELXS97* (Sheldrick, 2008 ▶); program(s) used to refine structure: *SHELXL97* (Sheldrick, 2008 ▶); molecular graphics: *SHELXTL* (Sheldrick, 2008 ▶); software used to prepare material for publication: *SHELXTL*.

## Supplementary Material

Crystal structure: contains datablocks global, I. DOI: 10.1107/S1600536808041457/ci2738sup1.cif

Structure factors: contains datablocks I. DOI: 10.1107/S1600536808041457/ci2738Isup2.hkl

Additional supplementary materials:  crystallographic information; 3D view; checkCIF report

## Figures and Tables

**Table 1:** Hydrogen-bond geometry (Å, °)

*D*—H⋯*A*	*D*—H	H⋯*A*	*D*⋯*A*	*D*—H⋯*A*
N1—H1⋯O2^i^	0.87	2.11	2.862 (2)	144
C19—H19*A*⋯*Cg*1^ii^	0.97	2.69	3.645 (3)	167

Symmetry codes: (i) 
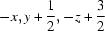
; (ii) 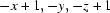. *Cg*1 is centroid of the C13–C18 ring.

**Fig. 1.**
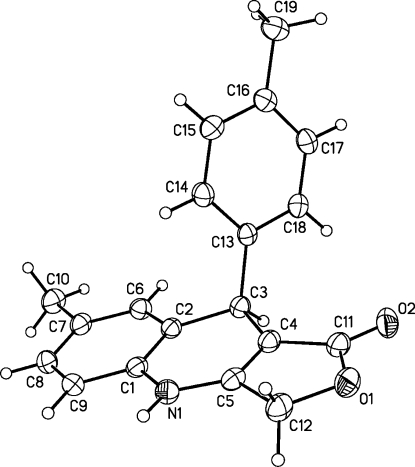
The molecular structure of the title compound, showing 30% probability displacement ellipsoids and the atom-numbering scheme.

**Fig. 2.**
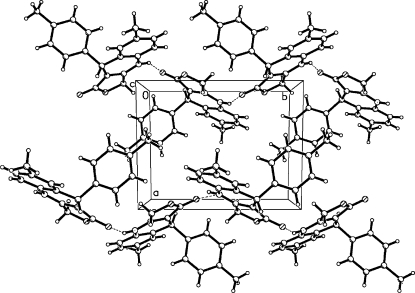
The crystal packing of the title compound. Hydrogen bonds are shown as dashed lines.

## supplementary crystallographic information

### Comment

Podophyllotoxin is an antitumor lignan that inhibits microtubule assembly (Eycken *et al.*, 1989; Tomioka *et al.*, 1989; Bosmans *et al.*, 1989). Because of mostly unsuccessfull attempts to use it for the treatment of human neoplasia and complicated side effects, extensive structural modifications have been performed in order to obtain more potent and less toxic anticancer agents (Tomioka *et al.*, 1993; Lienard *et al.*, 1991; Poli *et al.*, 2002). Among them, 4-aza-podophyllotoxin (9-aryl-4,9-dihydrofuro [3,4-*b*]quinolin-1(3*H*)-one) derivatives reported as powerful DNA topoisomerase II inhibitors, have recently attached considerable interest (Hitosuyanagi *et al.*, 1997; Hitosuyanagi *et al.*, 1999; Tratrat *et al.*,  2002; Magedov *et al.*, 2007). We report here the crystal structure of the title compound, which was synthesized by the three-component reaction of 4-methylaniline with 4-methylbenzaldehyde and tetronic acid catalyzed by *L*-proline using ethanol as solvent at 353 K.

In the title compound, the dihydropyridine ring (C1-C5/N1) adopts a flattened boat conformation, with atoms C3 and N1 deviating from the C1/C2/C4/C5 plane (r.m.s. deviation 0.009 Å) by 0.102 (3) and 0.050 (3) Å, respectively (Fig. 1). The five-membered ring is almost planar (r.m.s. deviation 0.018 Å). The dihedral angle between C1/C2/C6/C7-C9 and C1/C2/C4/C5 planes is 2.9 (1)° and that between C1/C2/C4/C5 and C13-C18 plane is 79.77 (7)°.

The molecules are linked into chains (Fig.2) along the *b* axis by N—H···O intermolecular hydrogen bonds (Table 1). In addition, C—H···π interactions involving the phenyl ring of the tolyl group are observed.

### Experimental

The title compound was prepared by the reaction of 4-methylaniline (1 mmol) and 4-methylbenzaldehyde (1 mmol) with tetronic acid (1 mmol) in the presence of *L*-proline (0.1 mmol) in ethanol (2 ml) at 353 K. Crystals of the title compound suitable for X-ray diffraction were obtained by slow evaporation of a *N*,*N*-dimethylformamide and ethanol solution. ^1^H NMR (DMSO-d_6_, δ): 2.12 (3*H*, s, CH_3_), 2.22 (3*H*, s, CH_3_), 4.85 (1*H*, d, J = 16.0 Hz, CH), 4.91 (1*H*, s, CH), 4.94 (1*H*, d, J = 16.0 Hz, CH), 6.80–6.84 (2*H*, m, ArH), 6.93 (1*H*, d, J = 8.0 Hz, ArH), 7.04–7.08 (4*H*, m, ArH), 9.94 (1*H*, s, NH).

### Refinement

H atoms were placed in calculated positions (N-H = 0.87 Å and C-H = 0.94–0.99 Å), and included in the final cycles of refinement using a riding model, with *U*_iso_(H) = 1.2–1.5 *U*_eq_(C). A rotating group model was used for the methyl groups.

### Crystal data

**Table:**

C_19_H_17_NO_2_	*F*(000) = 616
*M**_r_* = 291.34	*D*_x_ = 1.317 Mg m^−^^3^
Monoclinic, *P*2_1_/*c*	Mo *K*α radiation, λ = 0.71070 Å
Hall symbol: -P 2ybc	Cell parameters from 4940 reflections
*a* = 9.178 (2) Å	θ = 3.3–25.3°
*b* = 11.457 (2) Å	µ = 0.09 mm^−^^1^
*c* = 14.350 (4) Å	*T* = 223 K
β = 103.124 (5)°	Block, colourless
*V* = 1469.5 (6) Å^3^	0.60 × 0.48 × 0.45 mm
*Z* = 4	

### Data collection

**Table:**

Rigaku Mercury diffractometer	2675 independent reflections
Radiation source: fine-focus sealed tube	2364 reflections with *I* > 2σ(*I*)
graphite	*R*_int_ = 0.034
Detector resolution: 7.31 pixels mm^-1^	θ_max_ = 25.3°, θ_min_ = 3.3°
ω scans	*h* = −11→11
Absorption correction: multi-scan (Jacobson, 1998)	*k* = −13→12
*T*_min_ = 0.756, *T*_max_ = 0.962	*l* = −17→17
13947 measured reflections	

### Refinement

**Table:**

Refinement on *F*^2^	Primary atom site location: structure-invariant direct methods
Least-squares matrix: full	Secondary atom site location: difference Fourier map
*R*[*F*^2^ > 2σ(*F*^2^)] = 0.059	Hydrogen site location: inferred from neighbouring sites
*wR*(*F*^2^) = 0.135	H-atom parameters constrained
*S* = 1.16	*w* = 1/[σ^2^(*F*_o_^2^) + (0.0497*P*)^2^ + 0.6176*P*] where *P* = (*F*_o_^2^ + 2*F*_c_^2^)/3
2675 reflections	(Δ/σ)_max_ = 0.001
202 parameters	Δρ_max_ = 0.23 e Å^−^^3^
0 restraints	Δρ_min_ = −0.21 e Å^−^^3^

### Special details

**Table:**

Geometry. All e.s.d.'s (except the e.s.d. in the dihedral angle between two l.s. planes) are estimated using the full covariance matrix. The cell e.s.d.'s are taken into account individually in the estimation of e.s.d.'s in distances, angles and torsion angles; correlations between e.s.d.'s in cell parameters are only used when they are defined by crystal symmetry. An approximate (isotropic) treatment of cell e.s.d.'s is used for estimating e.s.d.'s involving l.s. planes.
Refinement. Refinement of *F*^2^ against ALL reflections. The weighted *R*-factor *wR* and goodness of fit *S* are based on *F*^2^, conventional *R*-factors *R* are based on *F*, with *F* set to zero for negative *F*^2^. The threshold expression of *F*^2^ > σ(*F*^2^) is used only for calculating *R*-factors(gt) *etc*. and is not relevant to the choice of reflections for refinement. *R*-factors based on *F*^2^ are statistically about twice as large as those based on *F*, and *R*- factors based on ALL data will be even larger.

### Fractional atomic coordinates and isotropic or equivalent isotropic displacement parameters (Å^2^)

**Table:**

	*x*	*y*	*z*	*U*_iso_*/*U*_eq_	
O1	−0.06775 (18)	0.28146 (14)	0.77022 (11)	0.0528 (4)	
O2	−0.09551 (18)	0.13853 (14)	0.66103 (12)	0.0549 (5)	
N1	0.14394 (19)	0.50270 (15)	0.68136 (12)	0.0396 (4)	
H1	0.1588	0.5617	0.7205	0.048*	
C1	0.1948 (2)	0.50585 (16)	0.59568 (14)	0.0343 (5)	
C2	0.1821 (2)	0.40751 (17)	0.53741 (13)	0.0329 (5)	
C3	0.1160 (2)	0.29193 (17)	0.56127 (14)	0.0342 (5)	
H3	0.0327	0.2709	0.5069	0.041*	
C4	0.0523 (2)	0.31008 (18)	0.64815 (14)	0.0366 (5)	
C5	0.0727 (2)	0.40748 (18)	0.70132 (14)	0.0374 (5)	
C6	0.2347 (2)	0.41594 (18)	0.45381 (14)	0.0382 (5)	
H6	0.2259	0.3505	0.4134	0.046*	
C7	0.2994 (2)	0.51643 (18)	0.42757 (15)	0.0400 (5)	
C8	0.3088 (2)	0.61316 (18)	0.48745 (16)	0.0424 (5)	
H8	0.3511	0.6827	0.4710	0.051*	
C9	0.2572 (2)	0.60876 (18)	0.57031 (16)	0.0401 (5)	
H9	0.2641	0.6750	0.6097	0.048*	
C10	0.3607 (3)	0.5198 (2)	0.33889 (16)	0.0513 (6)	
H10A	0.4686	0.5113	0.3565	0.077*	
H10B	0.3352	0.5938	0.3064	0.077*	
H10C	0.3178	0.4565	0.2966	0.077*	
C11	−0.0401 (2)	0.2330 (2)	0.68790 (16)	0.0440 (5)	
C12	0.0028 (3)	0.3941 (2)	0.78519 (16)	0.0479 (6)	
H12A	0.0788	0.3961	0.8455	0.058*	
H12B	−0.0709	0.4557	0.7860	0.058*	
C13	0.2291 (2)	0.19179 (16)	0.57700 (13)	0.0319 (4)	
C14	0.3781 (2)	0.20786 (18)	0.62247 (15)	0.0409 (5)	
H14	0.4124	0.2829	0.6429	0.049*	
C15	0.4770 (2)	0.11455 (19)	0.63815 (16)	0.0437 (5)	
H15	0.5776	0.1279	0.6687	0.052*	
C16	0.4311 (2)	0.00218 (17)	0.60993 (14)	0.0383 (5)	
C17	0.2828 (2)	−0.01293 (18)	0.56396 (15)	0.0425 (5)	
H17	0.2484	−0.0879	0.5433	0.051*	
C18	0.1838 (2)	0.07991 (17)	0.54770 (15)	0.0394 (5)	
H18	0.0837	0.0667	0.5161	0.047*	
C19	0.5382 (3)	−0.0990 (2)	0.62813 (17)	0.0511 (6)	
H19A	0.5792	−0.1118	0.5723	0.077*	
H19B	0.4856	−0.1686	0.6408	0.077*	
H19C	0.6187	−0.0820	0.6830	0.077*	

### Atomic displacement parameters (Å^2^)

**Table:**

	*U*^11^	*U*^22^	*U*^33^	*U*^12^	*U*^13^	*U*^23^
O1	0.0578 (10)	0.0565 (10)	0.0538 (10)	−0.0026 (8)	0.0326 (8)	0.0054 (8)
O2	0.0594 (10)	0.0468 (10)	0.0643 (11)	−0.0111 (8)	0.0259 (9)	0.0068 (8)
N1	0.0428 (10)	0.0394 (10)	0.0397 (10)	−0.0028 (8)	0.0160 (8)	−0.0071 (8)
C1	0.0297 (10)	0.0376 (11)	0.0370 (11)	0.0023 (8)	0.0104 (9)	0.0015 (9)
C2	0.0303 (10)	0.0354 (11)	0.0337 (10)	0.0012 (8)	0.0085 (8)	0.0035 (8)
C3	0.0328 (10)	0.0368 (11)	0.0343 (10)	−0.0020 (8)	0.0104 (8)	−0.0007 (8)
C4	0.0340 (10)	0.0408 (11)	0.0366 (11)	0.0017 (9)	0.0116 (9)	0.0047 (9)
C5	0.0313 (10)	0.0456 (12)	0.0370 (11)	0.0033 (9)	0.0111 (9)	0.0021 (9)
C6	0.0396 (11)	0.0398 (11)	0.0369 (11)	0.0026 (9)	0.0123 (9)	0.0015 (9)
C7	0.0359 (11)	0.0454 (12)	0.0400 (11)	0.0018 (9)	0.0114 (9)	0.0112 (9)
C8	0.0407 (12)	0.0365 (11)	0.0512 (13)	−0.0021 (9)	0.0129 (10)	0.0089 (10)
C9	0.0385 (11)	0.0340 (11)	0.0481 (12)	0.0002 (8)	0.0103 (10)	−0.0023 (9)
C10	0.0523 (14)	0.0589 (15)	0.0469 (13)	0.0007 (11)	0.0198 (11)	0.0130 (11)
C11	0.0407 (12)	0.0475 (13)	0.0478 (13)	0.0029 (10)	0.0184 (10)	0.0087 (10)
C12	0.0490 (13)	0.0545 (14)	0.0462 (13)	−0.0018 (10)	0.0232 (11)	−0.0011 (11)
C13	0.0363 (10)	0.0334 (10)	0.0283 (9)	−0.0030 (8)	0.0122 (8)	0.0000 (8)
C14	0.0415 (12)	0.0364 (11)	0.0433 (12)	−0.0054 (9)	0.0067 (9)	−0.0045 (9)
C15	0.0369 (11)	0.0479 (13)	0.0449 (12)	−0.0005 (9)	0.0065 (10)	−0.0002 (10)
C16	0.0470 (12)	0.0396 (12)	0.0316 (10)	0.0030 (9)	0.0158 (9)	0.0032 (8)
C17	0.0502 (13)	0.0344 (11)	0.0438 (12)	−0.0068 (9)	0.0123 (10)	−0.0035 (9)
C18	0.0368 (11)	0.0394 (11)	0.0416 (11)	−0.0067 (9)	0.0082 (9)	−0.0005 (9)
C19	0.0589 (14)	0.0472 (13)	0.0496 (13)	0.0105 (11)	0.0172 (11)	0.0042 (11)

### Geometric parameters (Å, °)

**Table:**

O1—C11	1.380 (3)	C8—H8	0.94
O1—C12	1.438 (3)	C9—H9	0.94
O2—C11	1.220 (3)	C10—H10A	0.97
N1—C5	1.336 (3)	C10—H10B	0.97
N1—C1	1.411 (3)	C10—H10C	0.97
N1—H1	0.87	C12—H12A	0.98
C1—C2	1.392 (3)	C12—H12B	0.98
C1—C9	1.395 (3)	C13—C18	1.383 (3)
C2—C6	1.395 (3)	C13—C14	1.387 (3)
C2—C3	1.528 (3)	C14—C15	1.387 (3)
C3—C4	1.506 (3)	C14—H14	0.94
C3—C13	1.529 (3)	C15—C16	1.386 (3)
C3—H3	0.99	C15—H15	0.94
C4—C5	1.341 (3)	C16—C17	1.382 (3)
C4—C11	1.430 (3)	C16—C19	1.504 (3)
C5—C12	1.494 (3)	C17—C18	1.384 (3)
C6—C7	1.386 (3)	C17—H17	0.94
C6—H6	0.94	C18—H18	0.94
C7—C8	1.393 (3)	C19—H19A	0.97
C7—C10	1.504 (3)	C19—H19B	0.97
C8—C9	1.377 (3)	C19—H19C	0.97
			
C11—O1—C12	108.95 (16)	C7—C10—H10C	109.5
C5—N1—C1	118.85 (17)	H10A—C10—H10C	109.5
C5—N1—H1	120.6	H10B—C10—H10C	109.5
C1—N1—H1	120.6	O2—C11—O1	118.87 (19)
C2—C1—C9	120.68 (18)	O2—C11—C4	131.6 (2)
C2—C1—N1	120.25 (17)	O1—C11—C4	109.52 (19)
C9—C1—N1	119.06 (18)	O1—C12—C5	103.48 (17)
C1—C2—C6	117.57 (18)	O1—C12—H12A	111.1
C1—C2—C3	123.37 (17)	C5—C12—H12A	111.1
C6—C2—C3	119.06 (17)	O1—C12—H12B	111.1
C4—C3—C2	108.32 (16)	C5—C12—H12B	111.1
C4—C3—C13	111.04 (16)	H12A—C12—H12B	109.0
C2—C3—C13	113.10 (15)	C18—C13—C14	117.52 (18)
C4—C3—H3	108.1	C18—C13—C3	120.23 (17)
C2—C3—H3	108.1	C14—C13—C3	122.24 (17)
C13—C3—H3	108.1	C13—C14—C15	120.86 (19)
C5—C4—C11	107.79 (19)	C13—C14—H14	119.6
C5—C4—C3	123.77 (18)	C15—C14—H14	119.6
C11—C4—C3	128.43 (19)	C16—C15—C14	121.6 (2)
N1—C5—C4	124.70 (19)	C16—C15—H15	119.2
N1—C5—C12	125.25 (19)	C14—C15—H15	119.2
C4—C5—C12	110.05 (19)	C17—C16—C15	117.18 (19)
C7—C6—C2	122.99 (19)	C17—C16—C19	121.35 (19)
C7—C6—H6	118.5	C15—C16—C19	121.5 (2)
C2—C6—H6	118.5	C16—C17—C18	121.46 (19)
C6—C7—C8	117.62 (19)	C16—C17—H17	119.3
C6—C7—C10	121.1 (2)	C18—C17—H17	119.3
C8—C7—C10	121.2 (2)	C13—C18—C17	121.37 (19)
C9—C8—C7	121.21 (19)	C13—C18—H18	119.3
C9—C8—H8	119.4	C17—C18—H18	119.3
C7—C8—H8	119.4	C16—C19—H19A	109.5
C8—C9—C1	119.9 (2)	C16—C19—H19B	109.5
C8—C9—H9	120.0	H19A—C19—H19B	109.5
C1—C9—H9	120.0	C16—C19—H19C	109.5
C7—C10—H10A	109.5	H19A—C19—H19C	109.5
C7—C10—H10B	109.5	H19B—C19—H19C	109.5
H10A—C10—H10B	109.5		
			
C5—N1—C1—C2	−5.5 (3)	C7—C8—C9—C1	0.3 (3)
C5—N1—C1—C9	174.32 (18)	C2—C1—C9—C8	−0.8 (3)
C9—C1—C2—C6	0.3 (3)	N1—C1—C9—C8	179.35 (18)
N1—C1—C2—C6	−179.82 (17)	C12—O1—C11—O2	−177.5 (2)
C9—C1—C2—C3	179.55 (17)	C12—O1—C11—C4	1.7 (2)
N1—C1—C2—C3	−0.6 (3)	C5—C4—C11—O2	174.9 (2)
C1—C2—C3—C4	7.1 (2)	C3—C4—C11—O2	−3.7 (4)
C6—C2—C3—C4	−173.67 (17)	C5—C4—C11—O1	−4.1 (2)
C1—C2—C3—C13	−116.4 (2)	C3—C4—C11—O1	177.31 (18)
C6—C2—C3—C13	62.8 (2)	C11—O1—C12—C5	1.1 (2)
C2—C3—C4—C5	−8.9 (3)	N1—C5—C12—O1	175.80 (19)
C13—C3—C4—C5	115.9 (2)	C4—C5—C12—O1	−3.7 (2)
C2—C3—C4—C11	169.54 (19)	C4—C3—C13—C18	93.7 (2)
C13—C3—C4—C11	−65.7 (3)	C2—C3—C13—C18	−144.25 (18)
C1—N1—C5—C4	4.0 (3)	C4—C3—C13—C14	−84.7 (2)
C1—N1—C5—C12	−175.41 (19)	C2—C3—C13—C14	37.3 (2)
C11—C4—C5—N1	−174.73 (19)	C18—C13—C14—C15	−0.4 (3)
C3—C4—C5—N1	4.0 (3)	C3—C13—C14—C15	178.10 (19)
C11—C4—C5—C12	4.8 (2)	C13—C14—C15—C16	−0.5 (3)
C3—C4—C5—C12	−176.55 (18)	C14—C15—C16—C17	1.0 (3)
C1—C2—C6—C7	0.7 (3)	C14—C15—C16—C19	−179.2 (2)
C3—C2—C6—C7	−178.55 (18)	C15—C16—C17—C18	−0.7 (3)
C2—C6—C7—C8	−1.2 (3)	C19—C16—C17—C18	179.6 (2)
C2—C6—C7—C10	177.29 (19)	C14—C13—C18—C17	0.7 (3)
C6—C7—C8—C9	0.7 (3)	C3—C13—C18—C17	−177.80 (18)
C10—C7—C8—C9	−177.79 (19)	C16—C17—C18—C13	−0.2 (3)

### Hydrogen-bond geometry (Å, °)

**Table:**

*D*—H···*A*	*D*—H	H···*A*	*D*···*A*	*D*—H···*A*
N1—H1···O2^i^	0.87	2.11	2.862 (2)	144
C19—H19A···Cg1^ii^	0.97	2.69	3.645 (3)	167

Symmetry codes: (i) −*x*, *y*+1/2, −*z*+3/2; (ii) −*x*+1, −*y*, −*z*+1.
